# Associations between Symptom Severity of Autism Spectrum Disorder and Screen Time among Toddlers Aged 16 to 36 Months

**DOI:** 10.3390/bs13030208

**Published:** 2023-02-27

**Authors:** Saeid Sadeghi, Hamid Reza Pouretemad, Reza Shervin Badv, Serge Brand

**Affiliations:** 1 Institute for Cognitive and Brain Sciences, Shahid Beheshti University, Tehran 19839-69411, Iran; 2 Department of Pediatric Neurology, Children’s Medical Center, Pediatrics Center of Excellence, Tehran University of Medical Sciences, Tehran 14166-34793, Iran; 3 Center of Affective, Stress and Sleep Disorders (ZASS), Psychiatric Clinics (UPK), University of Basel, 4002 Basel, Switzerland; 4 Division of Sport Science and Psychosocial Health, Department of Sport, Faculty of Medicine, Exercise and Health, University of Basel, 4002 Basel, Switzerland; 5 Sleep Disorders Research Center, Kermanshah University of Medical Sciences, Kermanshah 67158-47141, Iran; 6 Substance Abuse Prevention Research Center, Kermanshah University of Medical Sciences, Kermanshah 67158-47141, Iran; 7 School of Medicine, Tehran University of Medical Sciences, Tehran 14166-34793, Iran

**Keywords:** autism spectrum disorder, repetitive behaviors, screen time, social interaction

## Abstract

There is growing evidence that prevalence rates of autism spectrum disorder (ASD) are increasing. A number of factors appear to contribute to this increase, including excessive screen time. Screen time seems to be linked to the severity of the symptoms of ASD. Given this, the aim of the present cross-sectional study was to investigate the association between early screen time and ASD symptoms severity in the first 36 months of life. To this end, sixty-eight Iranian toddlers (mean age: 27.09 months; 22.1% females) with ASD were recruited. Parents completed the modified checklist for autism in toddlers (M-CHAT), the Repetitive Behavior Scale-Revised (RBS-R), and a lifestyle checklist. Next, parents rated children’s daily exposure to content specifically designed (foreground media) and not specifically designed (background media) for children, along with their daily exposure to social interaction. Per day, toddlers spent 5.12 h (±3.77) with foreground media, 3.72 h (±3.57) with background media, and 2.89 h (±2.74) in interaction with other people (parents). To test the hypotheses, we performed a series of Pearson’s correlations and multiple regression analyses. Toddlers’ higher severity scores for ASD symptoms were associated with longer foreground (r = 0.234, *p* = 0.001) and longer background (r = 0.180, *p* = 0.012) media duration, and with shorter duration of interaction with others (r = 0.192, *p* = 0.009). Toddlers spending 1 h more in foreground screen time and background screen time have 0.38 and 0.29 more units in the ASD symptom severity scale, respectively, while toddlers spending 1 h more in social interactions have 0.42 fewer units in the ASD symptom severity scale. The screen time and interaction duration are related to ASD symptoms severity of toddlers. The cross-sectional study design precludes causal associations, although bi-directional relationships appear plausible.

## 1. Introduction

Compared to other neurodevelopmental disorders, the prevalence rates of autism spectrum disorder (ASD) has increased within the last two decades [1], with a rise of up to 500% from 1992 to about 2000 [2]. According to the Salari and Rasoulpoor [3] study, the prevalence of autism spectrum disorders has increased worldwide. Nevertheless, the prevalence of ASD differs by country due to differences in awareness levels, cultural differences in how children’s behavior is interpreted, variability in screening tools, the lack of culture-sensitive diagnostic tools, the evaluation year, and the sample and study populations. Currently, one out of 54 children appears to show symptoms of ASD [4]. Several hypotheses have been proposed to explain the increased prevalence rates, such as expanded diagnostic criteria, increased awareness of ASD, earlier diagnosis, recognition that ASD is a lifelong condition, and alterations in lifestyle [5,6,7] (see also further details below). Further, descriptively, as the prevalence of ASD has increased, the exposure of young children to digital devices (smartphones, tablets, televisions, etc.) has increased in parallel [8,9]. It has been reported that parental screen time, parental attitudes, parents’ workload, and having a television in the bedroom, as well as the availability of digital devices, are the most important factors influencing children’s screen time [10,11].

### 1.1. Symptoms of ASD and Exposure to Screens

Associations between ASD and television viewing were further investigated. To illustrate, Waldman and Nicholson [12] concluded that early childhood television viewing was an important trigger for autism; about 38% of ASD diagnoses appeared to be attributable to an excess of television watching. Similarly, Hermawati and Rahmadi [13] observed a statistical association between early electronic screen exposure and autistic-like behaviors (e.g., language delay, short attention span, and hyperactivity) in nine children aged 44 to 78 months. A similar pattern of association was further observed in a child aged 5 years [14]. In a further cross-sectional study Heffler and Sienko [15] investigated the associations between early life social and digital media experiences and the development of autism spectrum disorder-like symptoms. Heffler and Sienko [15] found that, among 12-month-old toddlers, the combination of increased exposure to television or video or both and shorter caregiver-child interactive play duration were significantly associated with more significant ASD-like symptoms, but not with the risk of ASD. Chen and Strodl [16] investigated in their cross-sectional study the relationship between early electronic screen exposure and autistic-like behaviors among 29,461 preschoolers aged 0–36 months and observed that a longer screen time per day was associated with autistic-like behaviors at preschool age. Dong and Wang [17] examined the association between screen time and ASD symptoms in children with ASD. Results showed that a longer screen time correlated with a higher ASD severity and with limited language abilities. The causal associations between screen time and the development of ASD at three years was further investigated among a large cohort of 84,030 children [18], with the finding that a longer screen time at the age of one year predicted symptoms of ASD at the age of 3 among boys, but not among girls.

### 1.2. Theoretical and Evidence-Based Explanations for Symptoms of ASD and Exposure to Screens

Seven theories have been proposed to explain the associations between symptoms of ASD and exposure to screens.

First, over-engagement with screen-based media may cause social isolation and deprivation [19,20,21,22]. In this view, there is evidence that restricted environments and experiences and early social isolation can cause autism symptoms and repetitive behaviors. To illustrate, children growing up in institutions were at increased risk for “quasi-autistic” patterns of behavior [23,24], also labelled post-institutional autistic syndrome (PIAS) [25]. Furthermore, Pouretemad and Sadeghi [26] defined Post-Digital Nanning Autism Syndrome (PDNAS) as a condition in which young children develop subclinical autism symptoms due to excessive exposure to digital devices (for more than half of their waking hours). The researchers hypothesized that PDNAS is a new subtype of ASD caused by and associated with the children’s lifestyle and excessive screen time at an early age. In this case, it is assumed that young children’s autism symptoms are probably causally associated with the early excessive exposure to digital devices. Previous studies have indicated that social interaction is very important for toddlers with autism [27]. According to Sadeghi and Pouretemad [28], parent–child interactions decreased children’s autism symptoms and repetitive behaviors and normalized their electrophysiological characteristics. It seems that the increase in social interactions leads to the development of neural circuits of social behaviors and, as a result, this reduces the severity of autism symptoms. In the Dawson [29] model, early behavioral intervention that leads to adaptive interactions between the child and its environment reduces autism symptoms.

Second, a prolonged screen time may contribute to non-social neural circuits formation during critical periods of brain development and neuroplasticity, and a prolonged screen time may inhibit the development of neuronal social networks [15,30,31]. In accordance with this theoretical framework, excessively watching television appeared to unfavorably impact on the typical development of the visual cortex, hypothalamus/septum, and sensorimotor structures in children [32].

Third, interventional studies indicated that reducing young children’s screen time and promoting social interaction reduced autism severity and normalized brain electrophysiological patterns in children with ASD-like symptoms [28,33,34,35].

Fourth, while these theories indicated that excessive screen time appeared to be causally associated with higher symptoms of ASD, an inverse direction of influence is also highly conceivable; children, at a very early age, with socioemotional deficits appeared to be more likely to prefer screens to social interactions [36,37,38]. Indeed, among six-month-old toddlers, the diagnosis of ASD was associated with earlier and more intensive preference for and use of screens [39].

Fifth, children with ASD may also intuitively and deliberately use screens as a coping strategy for impulse control and response inhibition [40].

Sixth, and plausibly, difficult children’s behavior may encourage their parents to expose their children to more screen time as a coping strategy [41].

Seventh, and last, longer screen time was associated with higher sleep issues and feeding problems in young children with ASD [42]; in such cases, longer screen time exposure may be understood as a coping strategy.

### 1.3. The Present Study

As described above, research on the associations between screen time exposure and symptom severity of ASD has mainly assessed children of school age and adolescents. In contrast, such research is scarce among toddlers with ASD. Given this, the present study was the first to examine the relationship between screen time and the severity of ASD symptoms, before a toddler was formally diagnosed with ASD and before any standardized treatment intervention was implemented. Further, while previous research showed that background and foreground media have different effects among typically developing young children, such as reducing attention control [43], decreased inhibitory control [44], reduced cognitive flexibility [45], and decreased their ability to process verbal information [46], such fine-grained research on the association between background and foreground media and ASD among toddlers has received less attention so far. Note that the term ‘foreground programs’ refers to content specifically designed for children to actively draw their attention. Unlike foreground programs, ‘background programs’ refers to the situation where an older family member chooses a program that is not explicitly designed for children, or when the TV is left on but does not explicitly elicit the child’s attention [47].

With this background in mind, the following four hypotheses and one research question were formulated.

First, based on research other than in toddlers with ASD [16,17,18], we hypothesized that a longer exposure to screen time was associated with a higher symptom severity.

Second, based on research other than in toddlers with ASD [33,47,48,49,50], we hypothesized that a longer exposure to screen time was associated with a shorter duration of social interactions.

Third, based on research other than in toddlers with ASD [51,52,53], we hypothesized that a higher symptom severity was associated with a lower duration of social interactions.

Fourth, based on research other than in toddlers with ASD [43,44,45,46], we hypothesized that foreground and background screens impacted on children’s symptom severity.

The research question investigated the relationship between duration of foreground screens exposure, background screens exposure, and exposure to social interaction and toddlers’ symptom severity.

The present results have the potential to further understand the associations within a fine-grained analysis of screen exposure, social interaction, and symptom severity in toddlers, before a formally diagnosis of ASD and before starting a standardized treatment intervention.

## 2. Materials and Methods

### 2.1. Participants and Procedure

Parents of toddlers aged 16 to 36 months with a suspected diagnosis of ASD and referred to Tehran Autism Center (Tehran, Iran) between February 2021 and September 2022 were approached to participate in the present study. Eligible parents were fully informed about the aims of the study and the secure and anonymous data handling. Thereafter, parents signed a written informed consent form. Parents completed a booklet of questions covering sociodemographic information and their toddlers’ behavior (see details below). Next, experts in ASD performed a formal diagnosis based on the DSM-5 criteria [54]. The Diagnostic and Statistical Manual of Mental Disorders-fifth edition (DSM-5) is a guide to mental health and brain-related conditions and disorders, written, edited, reviewed, and published by the American Psychiatric Association (APA). The Ethics Committee of the Shahid Beheshti University (Tehran, Iran: code: SBU.ICBS 96/1020) approved the study, which was conducted following the seventh and current revision [55] of the Declaration of Helsinki. Inclusion criteria were as follows: 1. Toddlers under 36 months of age. 2. Both parents’ age was 18 years or older. 3. Parents expressed concerns that their toddler showed abnormal behavior and interactions. 4. Based on the DSM-5 criteria for ASD [54], a toddler was identified to suffer from ASD. 5. Parents signed a written informed consent form. 6. Children did not have genetic syndromes or somatic comorbidities such as hearing and visual deficits.

### 2.2. Formal Testing

All toddlers were evaluated by an ASD specialist with a Ph.D. in clinical psychology and at least one assistant (with at least a master’s degree in clinical psychology). The diagnosis was made based on informed clinical judgment, following interaction with the child, formal testing, and review of parent reports and records. Diagnoses were based on DSM-5 guidelines. Formal testing lasted between 90 and 120 min. Parents needed between 30 to 45 min to complete the questionnaires (see below).

### 2.3. Measures

#### 2.3.1. Sociodemographic Information

Parents reported their age (years), the number of children in the same family household, their highest educational level (high school; undergraduate; master degree; PhD/MD), and their current working status (working: yes vs. no). Parents reported their toddler’s age (months) and gender (male; female).

#### 2.3.2. Toddlers’ Symptom Severity of ASD

To assess toddlers’ symptom severity of ASD, parents completed the Farsi version [56] of the Modified Checklist for Autism in Toddlers (M-CHAT); M-CHAT [57] consists of 23 yes/no items. The M-CHAT is a simple measure to screen for toddlers’ symptoms of ASD between 16 and 30 months of age. Robins and Fein [57] present M-CHAT for clinical and research purposes. Higher scores indicate greater symptoms of ASD in the child. Validity and reliability of M-CHAT have been examined in Iranian children [56,58,59]. In this study, the internal consistency of the M-CHAT was demonstrated by a Cronbach’s alpha score of 0.78.

#### 2.3.3. Toddlers’ Repetitive Behavior

To assess toddlers’ repetitive behavior, parents completed the Farsi version [60,61] of Repetitive Behavior Scale-Revised (RBS-R) [62]: This scale is a 43-item parent rating scale that measures six dimensions of repetitive behavior (stereotyped behavior, self-injurious behavior, compulsive behavior, ritualistic behavior, sameness behavior, and restricted behavior). A higher score on RBS-R means repetitive behaviors are more severe. Lam and Aman [63] showed construct validity of subscales from 0.68 to 0.98 and internal consistency of subscales from 0.78 to 0.91. Most of these descriptions were reproduced from our previous studies using a similar scale [28,35,64]. The RBS-R has already been validated in the Iranian population [60,61]. In this study, the internal consistency of the RBS-R was demonstrated by Cronbach’s alpha scores for the subscales of between 0.76 and 0.88.

#### 2.3.4. Children’s Screen Time and Communication Duration

To assess the children’s screen time and communication duration, we compiled a checklist in which parents had to estimate the child’s screen time, interaction, and sleep. With this checklist, we measured the amount of children’s sleep, waking time, screen time, and interactions during a day. We calculated the average hours of children’s foreground and background screen time and communication. The total screen time was calculated by summing the foreground and background screen times. In addition, in this checklist, we collected demographic information about families and children. Most of the mothers, who are the main caregivers of children, were not working and were at home for the whole week. For this reason, we did not separate the weekdays and weekends in terms of the amount of screen time and interactions.

### 2.4. Statistical Analysis

First, to test the four hypotheses, we performed a series of Pearson’s correlations to investigate the associations between the toddlers’ age, scores for autism spectrum, repetitive behavior, screen time (foreground, background, total screen time) and the duration of interaction.

Second, to answer the research question, that is, to predict toddlers’ symptoms of ASD, multiple regression analyses were performed with toddlers’ symptoms of ASD as the dependent variable, and screen time (foreground, background, total time) and interaction duration as predictors. Following others [65,66], preliminary conditions to perform a multiple regression analysis were generally met, and as the sample size (*n* = 68) not very close to 100, the number of predictors × 10 should not be greater than sample size (here: 4 × 10 = 40 < 68); predictors should sufficiently explain the dependent variable (Rs and R^2^′s); and the Durbin Watson (DW) coefficient should be between 1.5 and 2.5, indicating that the residuals of the predictors were independent of each other (DW = 1.59). Last, the variance inflation factors (VIF) to test multicollinearity should be 1 < VIF < 10. In our model, the VIF was 3.09.

The level of significance was set at *p* < 0.05. All statistical computations were performed with SPSS^®^ 28.0 (IBM Corporation, Armonk, NY, USA) for Windows^®^.

## 3. Results

### 3.1. General Sociodemographic Information

Table 1 provides a descriptive statistical overview of the sociodemographic information. More than half of the parents had undergraduate degrees. The fathers were all employed, whereas the mothers were mostly housewives (86.8%). Most families only had one child (69.1%).

### 3.2. Correlations between Toddlers’ Scores for ASD and Repetitive Behaviors and Their Screen Time and Communication Duration

Table 2 provides a descriptive statistical overview and an overview of the Pearson’s correlation coefficients between toddlers’ symptoms of ASD and screen time (foreground, background, total screen time), and interaction time with others.

Toddlers’ higher symptoms of ASD were associated with higher foreground and background screen time and lower interaction duration. Toddlers’ higher foreground screen time was associated with their higher sameness and restricted behaviors. Toddlers’ higher background screen time was associated with their higher ritualistic and sameness behaviors. Additionally, toddlers’ higher repetitive behaviors (all subscales) were associated with a lower interaction duration.

### 3.3. Predicting Toddlers’ Symptoms of ASD

To predict toddlers’ symptoms of ASD, a multiple regression analysis was performed with toddlers’ symptoms of ASD and their’ screen time and interaction duration as predictors. Table 3 provides the statistical overview.

A toddler’s higher foreground, background, and total screen time, and a toddler’s lower interaction duration, predicted a toddler’s higher symptoms of ASD. When the amount of foreground screen time is increased by one hour, toddlers have 0.38 more units on the ASD symptoms severity scale. In addition, toddlers spending 1 h more in foreground screen time have 0.38 and 0.30 more units in sameness behaviors and restricted behaviors subscales of the RBS-R, respectively. In addition, ASD symptoms severity scores in toddlers increase by 0.29 units when background screen time is increased by 1 h. Toddlers spending 1 h more in background screen time have 0.30 and 0.41 more units in ritualistic behaviors and sameness behaviors subscales of the RBS-R, respectively. Furthermore, for 1 h decrease in the toddlers’ interaction duration, the toddlers’ ASD symptoms severity, stereotyped behaviors, self-injurious behaviors, sameness behaviors, compulsive behaviors and RBS-R total scores increased by 0.42, 0.49, 0.29, 0.30, 0.28, and 0.36 units, respectively.

## 4. Discussion

The aim of the present study was to investigate the associations between screen time and interaction duration of toddlers with ASD and their ASD symptom severity. The key findings were that toddlers’ higher symptoms of ASD were associated with their higher screen time duration and lower interaction duration. As such, the present results add to the current literature in the following four ways: First, among toddlers aged about 27 months, both a higher foreground and background screen time was associated with higher parent-rated symptoms of ASD. Second, and complementarily, a higher foreground and background screen time and higher parent-rated symptoms of ASD were associated with a shorter duration of social interactions. Third, a longer foreground and background screen time, and a shorter social interactional time, independently predicted parent-rated toddlers’ symptom severity of ASD. Fourth, and most importantly, it appears that such unfavorable patterns of associations were already observable among toddlers with a mean age of about 27 months.

Four hypotheses and one research question were formulated and each of these is considered below in turn.

### 4.1. Toddlers’ Longer Exposure to Screen Time and Higher Symptom Severity

Results showed a positive association of foreground, background, and total screen time with toddlers’ ASD symptoms severity. These correlations are consistent with our first hypothesis that a longer exposure to screen time is strongly associated with a higher ASD symptom severity. Furthermore, toddlers’ higher foreground screen time was associated with their higher sameness and restricted behaviors. Toddlers’ higher background screen time was associated with their higher ritualistic and sameness behaviors. These results are consistent with the results of Chen and Strodl [16], Dong and Wang [17] and Kushima and Kojima [18], which showed that a longer screen time was associated with higher ASD symptoms severity. To explain such a pattern of results, we rely on the theoretical framework of Heffler and Oestreicher [30], as follows: a prolonged screen time may contribute to ASD through the formation of non-social neural circuits during critical periods of brain development and neuroplasticity [15,30,31]. In addition to theories suggesting that prolonged exposure to digital devices can trigger ASD symptoms, theories in which the relationship is reversed may also explain this finding. For instance, Tamana and Ezeugwu [38], Montes [37], Tamana and Ezeugwu [38], and Tandon and Sasser [36] showed that very young children with socioemotional deficits appeared to be more likely to prefer screens to social interactions. Furthermore, Chonchaiya and Nuntnarumit [39] found among six-month-old toddlers that the diagnosis of ASD was associated with earlier and more intensive preference for and use of screens.

### 4.2. Toddlers’ Longer Exposure to Screen Time and Shorter Time of Social Interaction

Our second hypothesis was that a longer exposure to screen time was associated with a shorter duration of social interactions, and our data did confirm this assumption. Results showed that higher foreground and background screen time duration was strongly associated with lower interaction duration. The results are in line with previous studies in children, which reported that the more time children spent watching television, the less time they spent with others [22]. These findings can be explained by the fact that screen time usually replaces children’s social interactions and activities [49,50]. Further, the present findings are consistent with the theory that over-engagement with screen-based media may cause social isolation and deprivation [19,20,21,22].

### 4.3. Toddlers’ Higher Symptom Severity and Shorter Time of Social Interaction

Results showed that a higher symptom severity of ASD was associated with a shorter duration of social interactions. This confirms our third hypothesis, that the severity of ASD symptoms was associated with a shorter duration of social interactions [51,52,53]. Further, it appears plausible that a two-way relationship between the degree of interaction and the severity of autism symptoms should be considered. A child with ASD exhibits significant weaknesses in basic communication skills such as eye contact and joint attention. When a child scores high in ASD symptoms, other persons might be less likely to communicate with him or her. On the other hand, when parents score low in skills or time to communicate with their children, the child’s symptoms might become more pronounced [51,52].

The social motivation theory in autism [67] may further help to the explain the present pattern of results. The social motivation theory argues that children with ASD find social stimuli less rewarding than do children with typical development [68,69,70]. Based on this theory, children with ASD are less likely at an early age to pay attention to social information, such as faces and gaze directions, thereby limiting opportunities for social learning (e.g., less joint attention, collaborative play, fewer friendships), thus blunting social skill development [70]. In line with our findings, Chawarska and Macari [71] have shown that the attention of infants with ASD was more focused on background objects than on people. Furthermore, our results have a number of similarities with previous findings that indicated that encouraging young children’s social interaction reduced autism severity and normalized brain electrophysiological patterns in children with ASD-like symptoms [28,33,34].

### 4.4. Foreground and Background Screen Time and Toddler’s Symptom Severity

With the fourth hypothesis we assumed that foreground and background screen time impacted differently on toddlers’ symptom severity, and our data did confirm this. Results showed that there were significant correlations between foreground screen time (r = 0.38) and background screen time (r = 0.29), with severity of ASD, and with a stronger relationship with ASD severity for foreground screen time than background screen time. As such, the present pattern of results is in line with what has been observed elsewhere [43,44,45,46]. To explain such a pattern of results, previous research has suggested that the effects of digital devices on young children’s cognitive and psychosocial development may vary, depending on whether digital devices were used in the foreground or background [72]. From this perspective, a number of studies among typically developing infants and children have shown that foreground exposure negatively impacted on attention, cognitive abilities, and language skills [73,74,75], whereas background exposure negatively impacted on parent–child interactions, and the quantity and quality of play [49,50,76,77]. Foreground screens seem to have a greater “barrier effect” between a parent and a child, and such a barrier effect may result in higher scores for autism symptoms. The screen time ‘barrier effect’ means that a child’s exposure to screens appears to reduce the quality and quantity of parent–child communication [78].

### 4.5. Screen Time and Social Interaction Time to Predict Toddlers’ Symptom Severity

With the research question, we investigated the relationship between foreground screens exposure, background screens exposure, exposure to social interaction and a toddler’s ASD symptom severity. Results showed that foreground screen time, background screen time, total screens time, and social interaction duration predicted a child’s ASD severity. This pattern of results in in line with studies that showed that prolonged screen time in children with autism spectrum disorders negatively impacted on the development of children’s communication and social skills [17]. Such results further evidence that the environment has a significant effect on the clinical symptoms of children with ASD, in that a prolonged screen time appears to have an adverse effect on children’s social interaction and behavior regulation. Following Yousef and Eapen [79], a longer screen time was related to children’s higher degree of autism-like symptoms, and such a relationship was more apparent among younger children and those with more screen time. This is in line with our findings.

To explain such a pattern of results, the quality of the present data do not allow a conclusive neuronal, psychological or social-interactional answer. However, we advance the following five assumptions:

First, ASD is a neurodevelopmental disorder and, during the early stages after birth, the brain develops rapidly and is influenced both by internal neuronal developmental processes [80,81,82,83] and by external, and thus environmental, stimuli [84,85,86]. As a result, we hypothesize that the brain maturation of a child with ASD exposed to prolonged and excessive screen time might be slowed down in terms of less grey and white matter and ramification, fewer dendritic spikes, disrupted myelination, and higher levels of local functional connectivity and dysmaturation of interconnected regions responsible for processing higher-order social information.

Second, in contrast and in addition to the above mentioned assumptions and evidence, studies have shown that children with ASD may also intuitively and deliberately use screens as a coping strategy for their impulse control and response inhibition [40]. Nally and Houlton [87] investigated parental perceptions of television and video use of children with ASD. Their study found that TV was used to distract children with ASD and minimize behavioral outbursts. Lane and Radesky [88] assumed that children with ASD were experiencing more screen time, both because of the children’s social communication deficits, and because the content of children’s programs were more predictable; here, we note that individuals with ASD prefer precise anticipation, that is, the absence of unexpected changes, to unpredictability. In the similar vein, Thompson and Christakis [41] suggested that children with ASD may also use screen time to regulate their behavior.

The third assumption is that parents of children with ASD might expose their children to more screen time as a parent coping strategy to handle their children’s challenging social and interactional behavior.

The fourth assumption is that the child’s inherent neuronal development, the quantity and quality of external stimuli, the child’s coping preferences, and parents’ parenting styles and coping preferences do interact, so to increase the child’s ASD symptom severity. Again, the quality of the present data do not allow us to confirm such an assumption of interaction.

The fifth, last and, again, admittedly highly speculative assumption is that latent, unassessed and thus unknown factors may moderate the association between screen time exposure and a child’s ASD symptom severity.

### 4.6. Limitations and Future Directions

In spite of the novelty of the combination of predictors and sample group age, the following limitations need to be considered. First, the sample itself might be considered small, which can result in a low level of statistical power. However, our data convincingly demonstrated strong correlations between screen time and interaction duration among toddlers with ASD, thus 68 parents of toddlers with ASD constituted a homogeneous and reliable sample. Second, measurement issues must be addressed. To assess the child’s screen time and communication, we used a parent-rated, self-report lifestyle checklist; by their nature, subjective ratings might be biased. This holds particularly true as parents were fully informed about the purpose of the study, and such information might have triggered social desirability biases in the completion of the checklists. Third, clinical and psychiatric evaluations of parents’ and toddlers’ psychiatric concerns might have improved the quality of the data and the reliability of the pattern of results. Fourth, in this study screen time was measured overall. To measure screen time, it is better to distinguish between the type of device and the day of the week. This will help us better understand how digital devices affect autism symptoms. The fifth limitation relates to the toddlers’ age. The subjects we studied were very young. Often, autism diagnoses at this age are provisional and can be changed. Therefore, generalizing findings to other age groups should be performed with caution. Sixth, as comprehensively discussed above, the cross-sectional nature of the study design precludes causal interpretations. Given this, we propose that future studies should use longitudinal and interventional designs.

## 5. Conclusions

It can be concluded that, in toddlers, a higher level of foreground and background screen time is associated with symptoms of ASD and repetitive behaviors. Furthermore, a higher foreground and background screen time, as well as higher parent-rated symptoms of ASD, are associated with a shorter time of social interaction. It is conceivable that neuronal developmental characteristics, a child’s preferences for objects, a child’s preference for non-social coping styles, and parents’ parenting and coping styles with the social behavior of a child with ASD might independently and reciprocally increase a child’s ASD symptom severity. The exposure of young children to excessive screen time, regardless of the type of media, is a serious educational mistake. Parents often allow their children long screen time while they do various household chores, which may inhibit children’s social development. Toddler caregivers should manage screen time and provide natural opportunities for toddlers to develop their social skills and communication abilities.

## Figures and Tables

**Table 1:** Participants’ sociodemographic characteristics. The children did not attend daycare.

Age of Toddlers and Parents						
Variable	Sex	Scale	M	SD	Min	Max
Age of toddlers	Male (55)	Months	26.87	4.95	16	36
	Female (13)		28.00	4.71	20	36
Age of parents	Male	Years	33.46	4.95	24	43
	Female		38.10	4.09	30	47
Participants’ sociodemographic characteristics						
Variable				Parent	Frequency	Percent
Number of children in the family		One child		-	47	69.1
		Two children		-	19	27.9
		Three children		-	2	3
Education		High School		Father	7	10.3
				Mother	10	14.7
		Undergraduate		Father	41	60.3
				Mother	36	52.9
		Master		Father	14	20.6
				Mother	15	22.1
		Doctoral		Father	6	8.8
				Mother	7	10.3
Current job activity		Not working		Father	0	0
				Mother	59	86.8
		Working		Father	68	100
				Mother	9	13.2

Abbreviations: M, Mean: SD, Standard deviation: Min, Minimum: Max, Maximum.

**Table 2:** Overview of correlational computations (Pearson’s correlations) between toddlers’ symptoms of ASD and screen time (*n* = 68).

Variables		ASD	Screen-Time			Interaction	Repetitive Behaviors						
			Foreground	Background	Total		SB	SIB	RB	SAB	REB	CE	RBT
	ASD	1											
Screen-time	Foreground	0.38 **	1										
	Background	0.29 *	0.37 **	1									
	Total	0.41 **	0.84 **	0.82 **	1								
Interaction		−0.42 **	−0.49 **	−0.31 **	−0.49 **	1							
Repetitive behaviors	SB	0.58 **	0.21	0.26	0.29 *	−0.41 **	1						
	SIB	0.68 **	0.20	0.22	0.26 *	−0.29 *	0.78 **	1					
	RB	0.67 **	0.24	0.30 *	0.33 *	−0.23 *	0.79 **	0.87 **	1				
	SAB	0.66 **	0.35 **	0.41 **	0.47 **	−0.23 *	0.59 **	0.85**	0.80 **	1			
	REB	0.37 *	0.30 *	0.28	0.37 *	−0.28 *	0.64 **	0.59 **	0.78 **	0.73 **	1		
	CB	0.52 **	0.15	0.25	0.24	−0.28 *	0.70 **	0.73 **	0.69 **	0.60 **	0.62 **	1	
	RBT	0.42 **	0.16	0.29	0.28	−0.36 **	0.83 **	0.82 **	0.88 **	0.75 **	0.89 **	0.80 **	1
M	-	6.47	5.12	3.72	8.84	2.89	10.08	7.76	8.78	6.44	15.65	6.56	60.98
SD	-	(5.83)	(3.77)	(3.57)	(3.67)	(2.74)	(4.01)	(4.13)	(4.73)	(3.53)	(4.50)	(4.14)	(16.26)

Abbreviations: M-C, M-CHAT; SB, Stereotyped behaviors; SIB, Self-injurious behaviors; RB, Ritualistic behaviors; SAB, Sameness behaviors; REB, Restricted behaviors; CB, Compulsive behaviors; RBT, Repetitive behaviors total score. * Correlation is significant at the 0.05 level; ** Correlation is significant at the 0.01 level; M, Mean; SD, Standard deviation.

**Table 3:** Linear regression for variables predicting toddlers’ ASD symptoms severity.

Dependent Variable		Independent Variable	*R^2^*	*SE b*	β	*F*
Autism Symptoms Severity		Foreground screen time	0.15	0.18	0.38	11.26 ***
		Background screen time	0.09	0.19	0.29	6.18 *
		Total screen time	0.17	0.11	0.41	13.26 ***
		Interaction duration	0.17	0.22	−0.42	13.99 ****
Repetitive behaviors	Stereotyped behaviors	Foreground screen time	0.04	0.15	0.21	2.15
		Background screen time	0.07	0.16	0.26	3.38
		Total screen time	0.09	0.10	0.29	4.32 *
		Interaction duration	0.17	0.29	−0.41	9.13 **
	Self-injurious behaviors	Foreground screen time	0.04	0.15	0.20	2.14
		Background screen time	0.05	0.16	0.22	2.70
		Total screen time	0.07	0.09	0.26	3.85
		Interaction duration	0.09	0.23	−0.29	5.06 *
	Ritualistic behaviors	Foreground screen time	0.06	0.17	0.24	3.28
		Background screen time	0.09	0.18	0.30	5.13 *
		Total screen time	0.11	0.10	0.33	6.60 **
		Interaction duration	0.05	0.29	−0.23	2.91
	Sameness behaviors	Foreground screen time	0.12	0.12	0.35	7.20 *
		Background screen time	0.17	0.12	0.41	11.05 **
		Total screen time	0.22	0.07	0.47	14.62 ***
		Interaction duration	0.09	0.26	−0.30	5.13 *
	Restricted behaviors	Foreground screen time	0.09	0.17	0.30	4.43 *
		Background screen time	0.08	0.18	0.28	3.64
		Total screen time	0.13	0.11	0.36	6.76 *
		Interaction duration	0.08	0.38	−0.28	3.82
	Compulsive behaviors	Foreground screen time	0.02	0.15	0.15	1.21
		Background screen time	0.06	0.16	0.25	3.33
		Total screen time	0.06	0.09	0.24	3.18
		Interaction duration	0.08	0.24	−0.28	4.17 *
	RBS-R total score	Foreground screen time	0.03	0.65	0.16	1.19
		Background screen time	0.08	0.65	0.29	4
		Total screen time	0.08	0.40	0.28	3.82
		Interaction duration	0.13	1.35	−0.36	6.42 *

Abbreviations: * *p*  ≤  0.05, ** *p*  <  0.01, *** *p*  ≤  0.001, **** *p*  ≤  0.0001.

## Data Availability

All data are presented in the paper.
